# Regulation of glucose metabolism by p53: Emerging new roles for the tumor suppressor

**DOI:** 10.18632/oncotarget.389

**Published:** 2011-12-31

**Authors:** Esha Madan, Rajan Gogna, Madan Bhatt, Uttam Pati, Periannan Kuppusamy, Abbas Ali Mahdi

**Affiliations:** ^1^ Department of Biochemistry, Chhatrapati Shahuji Maharaj Medical University, Lucknow, India; ^2^ Transcription and Human Biology Laboratory, School of Biotechnology, Jawaharlal Nehru University, New-Delhi, India; ^3^ Department of Radiotherapy and Chemotherapy, Chhatrapati Shahuji Maharaj Medical University, Lucknow, India; ^4^ Dorothy M Davis Heart and Lung Research institute, Dept. of Internal Medicine, Ohio State University, Columbus, OH, USA

**Keywords:** p53, Metabolism, TIGAR, SCO2, Tumor Suppressor

## Abstract

p53 is well known as the “guardian of the genome” for differentiated and neoplastic cells. p53 induces cell-cycle arrest and cell death after DNA damage and thus contributes to the maintenance of genomic stability. In addition to this tumor suppressor function for pro-oncogenic cells, p53 also plays an important role as the central regulator of stress response by maintaining cellular homeostasis at the molecular and biochemical level. p53 regulates aerobic respiration at the glycolytic and oxidative phosphorylation (OXPHOS) steps via transcriptional regulation of its downstream genes TP53-induced glycolysis regulator (TIGAR) and synthesis of cytochrome c oxidase (SCO2). p53 negatively regulates glycolysis through activation of TIGAR (an inhibitor of the fructose-2,6-bisphosphate). On the contrary p53 positively regulates OXPHOS through upregulation of SCO2, a member of the COX-2 assembly involved in the electron-transport chain. It is interesting to notice that p53 antagonistically regulates the inter-dependent glycolytic and OXPHOS cycles. It is important to understand whether the p53-mediated transcriptional regulation of TIGAR and SCO2 is temporally segregated in cancer cells and what is the relation between these paradoxical regulations of glycolytic pathway with the tumor suppressor activity of p53. In this review we will elucidate the importance of p53-mediated regulation of glycolysis and OXPHOS and its relation with the tumor suppressor function of p53. Further since cellular metabolism shares great relation with the process of aging we will also try and establish the role of p53 in regulation of aging via its transcriptional control of cellular metabolism.

## ABSTRACT

p53 is well known as the “guardian of the genome” for differentiated and neoplastic cells. p53 induces cell-cycle arrest and cell death after DNA damage and thus contributes to the maintenance of genomic stability. In addition to this tumor suppressor function for pro-oncogenic cells, p53 also plays an important role as the central regulator of stress response by maintaining cellular homeostasis at the molecular and biochemical level. p53 regulates aerobic respiration at the glycolytic and oxidative phosphorylation (OXPHOS) steps via transcriptional regulation of its downstream genes TP53-induced glycolysis regulator (TIGAR) and synthesis of cytochrome *c* oxidase (SCO2). p53 negatively regulates glycolysis through activation of TIGAR (an inhibitor of the fructose-2,6-bisphosphate). On the contrary p53 positively regulates OXPHOS through upregulation of SCO2, a member of the COX-2 assembly involved in the electron-transport chain. It is interesting to notice that p53 antagonistically regulates the inter-dependent glycolytic and OXPHOS cycles. It is important to understand whether the p53-mediated transcriptional regulation of TIGAR and SCO2 is temporally segregated in cancer cells and what is the relation between these paradoxical regulations of glycolytic pathway with the tumor suppressor activity of p53. In this review we will elucidate the importance of p53-mediated regulation of glycolysis and OXPHOS and its relation with the tumor suppressor function of p53. Further since cellular metabolism shares great relation with the process of aging we will also try and establish the role of p53 in regulation of aging via its transcriptional control of cellular metabolism.

## INTRODUCTION

*p53*, often referred to as the “guardian of the genome,” is the most commonly mutated gene in human cancer [1]. In response to DNA damage or other types of stress, p53 acts as a sequence-specific transcription factor and orchestrates the appropriate cellular response by inducing cell-cycle arrest, apoptosis, senescence, or differentiation [2, 3]. Thus p53 mediates cell response to various cellular stressors and thus plays a pivotal role in tumorogenesis, cell-death and survival [4]. In cells experiencing DNA-damage, hypoxia, genotoxicity, cytotoxicity, and oxidative stress, p53 dissociates from its ubiquitin ligase MDM2, via various post-translational modifications which promote its stabilization and activation [5]. Active p53 migrates to the nucleus and activates the expression of its down-stream genes involved in cell-cycle regulation, DNA repair, senescence, and cell death [1]. Recent findings indicated that p53 plays an important role in the regulation of cellular metabolism, specifically glycolysis and OXPHOS in cancerous cells. Glucose metabolism is a major step in the development and sustenance of a variety of cancers in human body. However, how p53 regulates metabolism and the relation of this newly discovered role of p53 with its universal role of tumor suppressor is not known. The regulation of cellular metabolism determines the physiological response of cancer cells to nutrients and oxygen and accordingly chooses to promote cell-proliferation, growth and survival [6]. In this review, we will try to elucidate the complex network of p53 regulation of cellular metabolism and p53 tumor suppressor activity.

## P53 AND THE WARBURG EFFECT

Otto Warburg established that cancer cells undergo high rates of aerobic glycolysis due to lack of mitochondrial oxidative phosphorylation [7, 8]. This effect termed as the “Warburg effect” is one of the prime causes of malignancy. The cellular metabolism in cancer cells is dependent upon key cancer-related transcription factors, such as p53 and HIF-1 [8, 9]. p53 is now known to positively upregulate OXPHOS by transcribing SCO2 (a COX2 assembly protein) and p53 down-regulates glycolysis by transcribing TIGAR (an inhibitor of the fructose-2,6-bisphosphate) [8-11]. Recent research shows that loss of p53 enhances aerobic glycolysis in cancer cells, resulting in the development of a more aggressive and highly metastatic forms of cancer [12]. Most of the cancer forms are linked with a physiological phenomenon termed “hypoxia”, thus expression of HIF-1 plays an important part in the regulation of cancer-cell metabolism [13]. HIF-1 directly influences Warburg effect by transcriptional regulation of glycolytic enzymes such as HK2, PDK1, and LDH-A [8, 14]. Considering the importance of p53 in tumor suppression and the high mutation rate of p53 (>50%) in human tumors, recent findings suggest that the mutation of the p53 gene and the resultant loss of function of the p53 protein in tumors could be an important genetic change contributing to the Warburg effect.

## ROLE OF P53 IN REGULATING GLYCOLYSIS

p53 is now known to not only drive the damaged cells to undergo apoptosis but also coordinates how cells use nutrients to preserve their survival. The role of p53 in the negative regulation of cellular glycolysis is now reported [15]. The p53 downstream gene *TIGAR* (*T*P53-*i*nduced *g*lycolysis and *a*poptosis *r*egulator), is a direct transcriptional target of p53 and it alters the pathway in which cancer cells use cellular glucose. TIGAR shares functional sequence similarities with the bis-phosphatase domain (FBPase-2) of the bi-functional enzyme PFK-2/FBPase-2 (6-phosphofructo-2-kinase/fructose-2,6-bisphosphatase), which degrades fructose-2,6-bisphosphate (Fru-2,6-P_2_). Fru-2,6-P_2_ induces 6-phospho-1-kinase for conversion of fructose-6-phosphate to fructose-1,6-bisphosphate at the third step of the cellular glycolysis reaction. The decrease in the Fru-2,6-P_2_ level induces the formation of fructose-6-phosphate. TIGAR causes a significant reduction in the cellular Fru-2,6-P_2_ levels and thereby blocks glycolysis at this step. TIGAR directs the metabolism of cellular glucose through pentose phosphate pathway thus producing the redox-active reactive oxygen species (ROS) quenching NADPH [15]. TIGAR is direct transcriptional target of p53 and p53 has two binding sites in the TIGAR promoter named BS1 and BS2. BS1 shows little binding affinity towards p53 and lies upstream the +1 transcription start site, whereas BS2 shows strong binding affinity towards p53 and lies in the first intron downstream the +1 transcription start site [15]. Since TIGAR activates the pentose-phosphate pathway and causes an increase in the NADPH generation, this causes an increase in cellular glutathione (GSH) levels, which acts as ROS scavengers. Bensaad et al^12^ reported that overexpression of TIGAR cDNA in cancer cells led to ROS quenching and protection from p53-mediated apoptosis as a result of genotoxic stress and DNA-damage [16]. Further, it has been reported that TIGAR-mediated increase in the pentose-phosphate-pathway also led to substantial increase in the cellular DNA repair [17]. Increase in the cellular NADPH levels blocks the apoptotic effector caspases like caspase-2 and caspase-9 [18], thus resulting in neutralization of p53 apoptotic response.

ROS, generated under conditions of genotoxicity and mitochondrial stress, transcriptionally activate p53, facilitate its nuclear entry, and inhibits p53 binding with MDM2 [19]. It appears that the sequence of cellular events involving activation of p53, TIGAR upregulation, inhibition of glycolysis, increase in pentose-phosphate pathway, generation of ROS scavenging GSH and NADPH are a part of the p53 feedback loop to neutralize ROS-induced cell death. Further, this pathway might help in DNA and cellular damage repair in cancer cells suffering from repairable dose of cellular/genotoxic insult [15]. Interestingly, the mechanism behind the choice made by p53 to drive cells for apoptosis or to facilitate cellular repair through TIGAR pathway is unknown. Collectively, these observations suggest that the effects of p53 on metabolism are responses to environmental conditions.

## ROLE OF P53 IN THE REGULATION OF OXIDATIVE PHOSPHORYLATION

Oxidative phosphorylation (OXPHOS) is the mitochondrial pathway of energy generation which succeeds tricarboxylic acid (TCA) cycle to produce adenosine triphosphate (ATP) through electron transport-coupled OXPHOS [6]. p53 is now shown to directly regulate OXPHOS in mice and human cancer cell lines through transcriptional upregulation of its downstream gene synthesis of cytochrome *c* oxidase (SCO2) [10]. p53 directly binds to SCO2 promoter and induces an increase in its mRNA and protein level. SCO2 is a COX assembly protein required for transfer of copper to the cytochrome *c* oxidase (COX) complex. COX, the inner mitochondrial membrane protein comprises of thirteen subunits (three mitochondrial-encoded subunits and ten nuclear-encoded subunits). SCO proteins (SCO1 and SCO2) are part of the COX holo-enzymes and are required for transfer of copper from COX17 to COX [20]. SCO2 acts upstream of SCO1 in this pathway, and is indispensable for COX II synthesis [21, 22]. *SCO1* and *SCO2* gene mutations in humans results in poor formation of COX and COX deficiency. *SCO2* mutations result in neonatal encephalo-cardiomyopathy, spinal muscular atrophy (SMA), neonatal hepatic failure, and fatal hypertrophic cardiomyopathy [23, 24]. Most of the SCO2 patients carry an E140K missense mutation on one allele adjacent to the conserved CxxxC motif which regulates the efficiency of SCO2 to bind to copper and function as a redox protein [22].

SCO2 protein is regulated by p53 thus it can be postulated that p53 via SCO2 plays a major role in copper homeostasis. Copper is stored in the mitochondrial matrix space and mitochondrial inner membrane is impermeable to copper transport, thus copper-binding membrane proteins are indispensable to the transport of copper between the mitochondria and the cytoplasm. In fact, severe cellular copper deficiency is observed in patients with non-functional SCO2 protein and further wild-type SCO2 overexpression complements the copper-deficiency phenotype [25]. The transcriptional regulation of SCO2 by p53 might also be responsible for p53-mediated regulation of mitochondrial signals and cellular thiol–disulfide oxidoreductase reactions required for oxidation of the copper-binding cysteine amino acids in the mitochondrial proteins [22].

In human colon cancer cell lines, DLD1 and SW480, the overexpression of SCO2 protein increase OXPHOS even in presence of p53 mutations suggesting that p53-mediated regulation of OXPHOS is via SCO2 [10]. In HCT116 human colon cancer cell line, deficiency in p53 causes low expression of SCO2, resulting in lower OXPHOS which is balanced by the increase in glycolysis [26]. This suggests that the downregulation of p53-dependent regulation of SCO2 impairs the mitochondrial respiratory chain, causing a shifting of ATP production from OXPHOS to glycolysis.

## RELATIONSHIP BETWEEN TIGAR, SCO2 AND P53-MEDIATED EFFECTOR RESPONSES

TIGAR suppresses glycolysis which generates necessary NADPH required for the OXPHOS. Thus synthesis of TIGAR should down-regulate OXPHOS, instead p53 transcriptionally synthesizes another gene SCO2 which up-regulates OXPHOS. It is understandable that in order to support p53 and SCO2-mediated increase in OXPHOS the cellular glycolytic levels must also be high. Thus p53 cannot transcriptionally regulate TIGAR and SCO2 at the same time and their regulation via p53 must be temporally segregated. It is logical to believe that p53 might upregulate either TIGAR or SCO2 at one point in time depending upon the intensity of the cellular and genotoxic stressors; however no data is available to support this hypothesis. On basis of the existing literature and the cellular outcomes observed upon upregulation of TIGAR and SCO2 in cancer cells, the cellular conditions which might lead to the synthesis of these genes can be predicted. Further, the individual role of TIGAR and SCO2 in assisting the tumor suppressor response of p53 can be hypothesized.

The effect of TIGAR on cell survival was proposed to be cell and context dependent [15]. Interestingly, the switch from p53-induced cell-cycle arrest to apoptosis following prolonged stress is associated with a decrease in expression of both TIGAR and p21^WAF1/CIP1^, suggesting that the induction of the apoptotic response may reflect upon the loss of protection by these p53-inducible survival signals. TIGAR might be recruited by p53 to induce cell-cycle arrest in cancer cells undergoing mild cellular and genotoxic stress. Most importantly TIGAR has the ability to reduce cellular ROS, which is necessary for the p53-mediated apoptotic response. TIGAR lowers glycolysis and thus significantly reduces the cellular ATP level, which is crucial for cell division and other housekeeping activities. We predict that TIGAR falls into the group of genes that are activated by low levels of stress and plays a key role in the tumor-suppressor function of p53. p53 tumor suppressor provides an opportunity of survival and resurrection to the cancer cells which are suffering for repairable doses of cellular/genotoxic insult through upregulation of TIGAR protein. A model has been proposed which explains the putative mechanism of the p53-mediated transcriptional regulation of TIGAR in inducing the cell-cycle arrest response in cancer cells (Figure 1a).

**Figure 1a:**
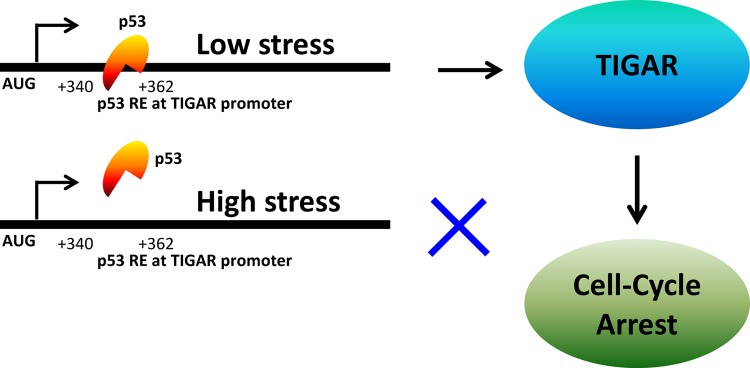
A model depicting the mechanism of p53-mediated regulation of TIGAR gene in cancer cells

SCO2, on the other hand, increases OXPHOS and supports cell division and most importantly increase the ATP supply to the cancer cells. It is a well-known fact that the cellular apoptosis is an ATP-dependent process [27]. Further, reactive oxygen species (ROS) is a toxic by-product of the mitochondrial energy production pathway, oxidative phosphorylation (OXPHOS), in cancer cells [28]. Since p53 increases OXPHOS via SCO2, it is logical to assume that p53-mediated SCO2 upregulation will eventually lead to accumulation of cellular ROS. ROS plays a major role in the progressive accumulation of cellular and tissue damage in neoplastic cells [29]. Further ROS also provides an efficient pathway of eliminating cancerous cells through apoptosis [29]. In addition to the synthesis of ROS there might be other pathways which determine the SCO2- and p53-mediated apoptotic response in the cancer cells which are suffering for the non-repairable doses of cellular/genotoxic stress. A model has been proposed to elucidate the predicted mechanism of p53-mediated transcriptional regulation of SCO2 gene in cancer cells (Figure 1b). It has become clear that in addition to the regulation of cellular metabolism, the p53-mediated transcriptional regulation of TIGAR and SCO2 is another tool in the armory of p53 to choose and protect cells which are capable of repair and further to effectively drive the non-repairable cells towards cellular apoptosis.

**Figure 1b:**
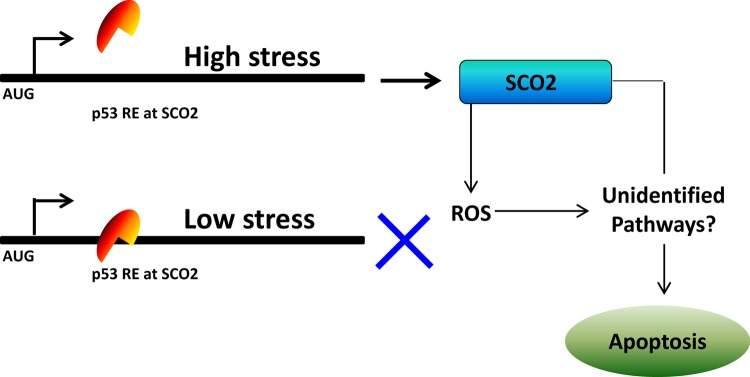
A model depicting the mechanism of p53-mediated regulation of SCO2 gene in cancer cells

## ROLE OF P53 IN METABOLISM AND AGING

The metabolic rate of organisms is related with their aging process [30]. Aging is dependent and proportional to the generation of ROS, a byproduct of cellular metabolism, which attack cell constituents and cause damage to cellular structures, and ROS is a by-product of metabolic reactions. ROS can induce apoptosis in cancer cells [19] thus cancer cells try and neutralize cellular oxidative stress via activation and upregulation of metabolic machinery involved in glycolysis. The crucial steps of this metabolic response against ROS production involves activation of the (i) pentose phosphate pathway (PPP), which regulates the redox status via the NADP+/NADPH ratio and (ii) glutathione system [7, 31].

Both anaerobic and aerobic glycolysis forms are high in cancer cells [8]. p53 is also now linked to regulate cellular aging through its newly found role in cellular metabolism. In cancer cells, p53-signaling molecule AMPK acts as an energy sensor and increases glycolysis [32, 33]. AMPK can also activate the tumor suppressor p53 in a feed-back loop, thus promoting cellular survival and aging [34]. Recent studies by Kawauchi et al. [35, 36] have revealed that increased glycolysis can enhance the O-glycosylation of IKKβ that subsequently triggers the NF-κB signaling. Growing literature shows that the IKK/NF-κB signaling pathway is involved in the carcinogenesis [37-39] but many observations also demonstrate that NF-κB system is activated during the aging process [40]. These observations refer to the fundamental role of glycolysis in cancer cells but it raises the question of whether p53 and the glycolytic flux can also regulate NF-κB signaling during the aging process. Interestingly, Kawauchi et al. [35] observed that the glycolysis-dependent activation of NF-κB signaling is regulated by tumor suppressor p53 oncogene.

Since p53 has a profound effect on metabolism and it increases aerobic metabolism and inhibits glycolysis [41]. The O-glycosylation of NF-κB at its Ser/Thr residues via HBP pathway has revealed the role of p53, NF-κB and metabolism in the process of aging [35, 36, 42]. p53 is now known to negatively regulate the IKK/NF-κB signaling through p53-dependent regulation of glycolysis via TIGAR. High glucose levels increase O-GlcNAc of IKKβ at its Ser733 residue, which is an inactivating phosphorylation site of the enzyme [35, 36]. High glucose levels can also increase the O-GlcNAc modification of p65, the trans-activating component of the NF-κB complex [43]. O-GlcNAc-p65 does not bind to its inhibitory protein Iκ-Bα and thus increases NF-κB-mediated transcription. Thus regulation of cellular glucose levels and glycolysis by p53 controls the NF-κB-mediated transcription. The p53-mediated regulation of TIGAR seems to function in another feed-back loop since O-GlcNAc-p53 is protected from its ubiquitination-mediated degradation [44]. p53 deletions and mutations will result in transcriptional inhibition at TIGAR promoter, thus an increases the glycolysis and the transcriptionally active O-GlcNAc- NF-κB thus the development and progression of cancer [39]. It is logical to assume that p53 might suppress the NF-κB signaling via the inhibition of glycolysis through regulation of TIGAR protein. NF-κB signaling and glycolysis are involved in pre-mature aging. The anti-aging proteins and the p53 c-terminus de-acetylases Sirtuin family of proteins, SIRT1 and SIRT6, are powerful inhibitors of NF-κB signaling as well. It is established that p53 lies at the nexus of the phenomena of aging and cancer [45, 46]. p53 stabilization in mouse models have resulted in resistance to cancer in combination with the development of the signs of pre-mature aging [47]. P53 transcriptional activity also decreases with increasing age of animals [48]. It is believed that decrease in p53 transcriptional activity in mammalian systems will lead to increase in glycolysis and decrease in OXPHOS, ultimately resulting in a strong relation between metabolism and aging.

## P53 AT THE NEXUS OF CELLULAR AGING AND SENESCENCE

Cellular senescence is a complexity of aging and cancer biology. As malignant tumors can culminate individuals in life-termination, evolution has provided multicellular organisms with an acquisition of distinctive safety device, cellular senescence, to circumvent the development. Cellular senescence, a state of irreversible growth arrest and cellular aging, an increase in the probability of death over survival, both can be triggered by multiple mechanisms. Eukaryotic cells have carefully devised these checkpoints to limit oncogenic cellular proliferation. Senescent cells may remain viable and quiescent in culture conditions and in the organisms [49] or may be cleared by the phagocytic machinery [50]. Senescence and aging are linked since aging is accompanied by increase in the volume of senescent cells and decrease in the division/regeneration potential of the stem-cell pool [51]. The telomere clock is one connecting link between senescence and aging. Telomere-mediated protection against cancer induces senescence and/or apoptosis [52, 53]. Telomere-induced senescence is now an established anti-neoplastic mechanism [54]. In humans, an inverse correlation between telomere length and cellular aging and between telomere length and aging related diseases is now well established [55, 56]. Factors which are known to decrease longevity, are known to decrease telomerase activity and telomere length [57, 58]. Furthermore, human premature aging syndromes, like dyskeratosis congenita and aplastic anemia, are linked with telomere shortening [59]. Further reinforcing the link between the telomere clock and aging is the observation that mice deficient in telomerase activity have short telomeres and age prematurely [60, 61]. Remarkably, even the first generation of telomerase-deficient mice already has a shortened lifespan, which becomes shorter and shorter in subsequent generations [62]. Moreover, telomeres become measurably shorter in mice, particularly at very old ages [63]. Finally, mice overexpressing telomerase are prone to developing tumors [64, 65], thus precluding direct demonstration of lifespan extension by telomerase; nonetheless, an increase in the lifespan of those few telomerase transgenic mice that do not develop cancer has been observed [66]. p53 plays a critical role in maintaining telomere stability [67]. p21Cip1, a direct transcriptional target of p53, has been shown to be relevant in the signaling of critically short telomeres that leads to aging [68]. p21Cip1 gene deletion in mice models has been shown to increase longevity of telomerase-deficient mice. Further no tumor formation was observed which was paradoxical to the effects observed in p53-deficient mice [69]. This data further confirmed that p53/p21Cip1-dependent senescence specifically induces the pro-aging effect of short telomeres. It is now believed that p53-mediates both the pro-aging function of telomere shortening [70].

The role of p53 in senescence and aging is not clearly established. Recently several transcription factors have been identified which can both inhibit senescence and induce epithelial-mesenchymal transition (EMT) thereby increasing the probability of cancer development [71]. Mice programmed to constitutively overexpress transcriptionally active mutant p53 have minimal probability of tumor development than wild-type mice. However, such programmed mice show signs of premature aging [72, 73]. Interestingly, mice with increased wild-type p53 activity do not exhibit premature aging. Super-p53 mice, carrying an extra copy of the entire p53 gene, show minimal probability of tumor development teamed up with a display of normal longevity [74]. Extra dose of p53 also increases the antioxidant function of p53 [16, 75]. The conclusion from these observations can be made which is suggestive of the fact that the main role of p53 is to eliminate damaged cells, either by triggering their self-destruction (via apoptosis) or by stalling them out of the proliferative pool (via senescence). During repairable doses of cellular insult, p53 induces the cell-repair pathways and thus activates the anti-aging program which compensates for the pro-aging response evoked by p53-mediated apoptosis. Exogenous addition of p53 copy can cause reversible cell-cycle arrest and/or irreversible senescence. Nutlin-3a (a small molecule that activates p53 without causing DNA damage), is unique in inducing senescence without making the cells quiescent. Interestingly, nutlin-3a inhibits the mTOR (mammalian target of rapamycin) pathway, a known member of the senescence program. It is recently shown that the negative regulators of the mTOR signaling, partially converts quiescence into senescence in the nutlin-treated cells. The mTOR pathway thus is one of the determining factors in facilitating the choice of p53 between the senescence and quiescence program in the p53-arrested cells [76, 77]. Further it has been shown that the p53-induced quiescence is in fact a result of suppression of senescence by p53. In cancer cells arrested with overexpression of p21, the addition of p53 converted senescence into quiescence and this suppression of senescence by p53 was dependent on p53-transcriptional activity. Thus, in spite of its ability to induce cell-cycle arrest, p53 can act as a suppressor of cellular senescence [33, 78]. p53 can promote or retard aging, depending on the context of its regulation and activity. p53 can regulate both pro-aging and pro-longevity effects by either inducing excessive apoptosis/cellular senescence or by eliminating damaged or dysfunctional cells via apoptosis/senescence [79]. p53 also transcriptionally regulates insulin/insulin-like growth factor (IGF)-1 involved in the IIS pathway. IIS and one of its major intracellular targets, the mTOR pathway, drive aging in mice [80]. In general, high IIS/mTOR activity is associated with cell proliferation, growth and aging, whereas low IIS/mTOR activity is associated with somatic maintenance and longevity. In addition, p53 is regulated, directly and indirectly (through MDM2), by another major component of IIS signaling, the PKB/Akt kinase [81]. PKB/Akt signaling in turn is also both pro-aging (through the NF-kB transcription factor) and pro-longevity (through FOXO transcription factors) [82]. p53 transcriptional activity depends mainly on post-translational modifications and protein/protein interaction [83]. Even though cancer cells have partially lost the capacity to signal senescence or apoptosis, these responses can conceivably be engaged by exogenous agents like p53-activating drugs and p53-modulating molecular chaperones [84-86].

The p53-dependent association between cellular senescence and organismal aging is highly suggestive of a causal link between these two processes. The cellular factors which trigger senescence also trigger apoptosis and cell-cycle arrest which makes it challenging to distinguish between the role of these three processes in aging [87]. It is interesting to observe that cancer is more frequent in old age human beings. The simplest explanation for this is that old organisms have accumulated more genetic and epigenetic aberrations than young organisms, but this does not say anything about the intrinsic susceptibility (that is, apart from the accumulation of mutations) of young and old organisms to develop cancer. Higher incidence of cancer at old ages simply reflects the time needed for the accumulation of oncogenic mutations. However, one must not neglect that with higher age there is a convenient environment for cancer growth. Great progress has been made in the last few years in understanding how cells limit their proliferative potential and in linking these mechanisms to cancer protection and aging. Future research will clarify whether therapies that induce senescence are useful for cancer treatment, and determine their effect on aging. Conversely, treatments that inhibit senescence in healthy individuals might slow aspects of aging.

## CONCLUSION AND FUTURE DIRECTIONS

In summary, p53's myriad of functions is beyond the tumor suppressor roles of being a protein involved in cell-cycle arrest, apoptosis and DNA-repair. However, the regulation of p53 has turned out to be highly complex and context-dependent. For instance, emerging results suggest that p53 has both nuclear and cytoplasmic functions [88] which may explain some of the controversial observations in transgenic models. p53 is now known to regulate stress-induced transcriptional programs that manipulate the energy metabolism of cancer cells in order to facilitate the tumor suppressor role of p53. The mechanisms regulating the opposite cell fates in cancer development and organism aging have been exciting cell biological topics for years. In particular, a lot of research effort has been focused on the role of p53 in the regulation of longevity [89, 90]. Furthermore, the role of p53 in the regulation of energy metabolism is a promising research field [41] which can provide some explanations to the puzzles of cancer and aging. It is established that energy metabolism is crucial for ever-proliferating cancer cells for their continued growth and survival. The activation of p53 to alternatively regulate the two most important aspects of metabolism, glycolysis via TIGAR and OXPHOS via SCO2 reveals that there are functions for p53 in the regulation of other metabolic diseases. Interestingly, how p53 can antagonistically regulate two crucial steps of the respiration cycle and the relation of this regulation with the well-defined tumor suppressor role of p53 needs to be elucidated.
